# Predicting the growth of the amphibian chytrid fungus in varying temperature environments

**DOI:** 10.1002/ece3.8379

**Published:** 2021-12-17

**Authors:** Zachary Gajewski, Lisa A. Stevenson, David A. Pike, Elizabeth A. Roznik, Ross A. Alford, Leah R. Johnson

**Affiliations:** ^1^ Department of Biological Science Virginia Tech Blacksburg Virginia USA; ^2^ Department of Statistics Virginia Tech Blacksburg Virginia USA; ^3^ 8001 College of Science and Engineering James Cook University Townsville Qld Australia; ^4^ 8001 North Carolina Zoo Asheboro North Carolina USA

**Keywords:** amphibian chytrid fungus, Bayesian hierarchical model, fluctuating temperatures, thermal ecology, thermal performance curves

## Abstract

Environmental temperature is a crucial abiotic factor that influences the success of ectothermic organisms, including hosts and pathogens in disease systems. One example is the amphibian chytrid fungus, *Batrachochytrium dendrobatidis* (*Bd*), which has led to widespread amphibian population declines. Understanding its thermal ecology is essential to effectively predict outbreaks. Studies that examine the impact of temperature on hosts and pathogens often do so in controlled constant temperatures. Although varying temperature experiments are becoming increasingly common, it is unrealistic to test every temperature scenario. Thus, reliable methods that use constant temperature data to predict performance in varying temperatures are needed. In this study, we tested whether we could accurately predict *Bd* growth in three varying temperature regimes, using a Bayesian hierarchical model fit with constant temperature *Bd* growth data. We fit the Bayesian hierarchical model five times, each time changing the thermal performance curve (TPC) used to constrain the logistic growth rate to determine how TPCs influence the predictions. We then validated the model predictions using *Bd* growth data collected from the three tested varying temperature regimes. Although all TPCs overpredicted *Bd* growth in the varying temperature regimes, some functional forms performed better than others. Varying temperature impacts on disease systems are still not well understood and improving our understanding and methodologies to predict these effects could provide insights into disease systems and help conservation efforts.

## Graphical abstract

We use a hierarchical Bayesian model fit with constant temperature data to predict growth of the amphibian chytrid fungus. We compare how using different thermal performance curves to constrain our temperature‐dependent parameter affects predictions. We find that we overpredict the growth of the amphibian chytrid fungus in three different varying temperature regimes, and different thermal performance curves strongly impact predictions.

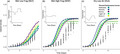

## 1 INTRODUCTION

Temperature is often a key factor in determining whether an ectothermic organism can succeed in a particular environment. Temperature affects multiple characteristics and traits of ectotherms, such as development rates, reproduction, and behavior, that impact the success of individuals or populations in an environment (Cator et al., 2020; Johnson et al., 2016; Lemoine et al., 2013; Nielsen & Papaj, 2015). Therefore, it is not surprising that temperature effects on organisms have been studied in many different systems.

Studies that examine temperature effects on organisms often do so in the laboratory under highly controlled conditions where temperature is set to one or multiple constant levels for the duration of an experiment. Many of these studies examine an organism's performance at several temperatures and compare the results at different conditions (Adamo & Lovett, 2011; Bieri et al., 1983; Damos & Savopoulou‐Soultani, 2008; Fielding & Ruesink, 1988; Stevenson et al., 2013; Voyles et al., 2017). Such studies have been used to answer questions about temperatures that optimize performance/traits, the thermal ranges of traits, and thermal adaptation of traits.

One way that the results of these thermal experiments are compared or summarized is by fitting a thermal performance curve (TPC) to the performance data across temperature treatments. TPCs are mathematical functions that are used to describe how an organism performs over a range of temperatures (Huey & Stevenson, 1979). Most of these functions are assumed to be unimodal, meaning that they exhibit an optimal temperature ( $T_{\mathrm{opt}}$) where the trait being described is maximized. These functions also typically include maximum and minimum temperature thresholds denoted as $T_{\max}$ and $T_{\min}$, respectively, where the trait being measured approaches, or is equal to, zero (Angilletta Jr, 2006; Huey & Kingsolver, 1989). TPCs have been fit for numerous species across a variety of traits using constant temperature data (Deutsch et al., 2008; Niehaus et al., 2012; Stevenson et al., 2013; Voyles et al., 2017). These functions describe how performance changes over constant temperatures and can be used to infer performance at temperature not directly measured (Huey & Stevenson, 1979).

Thermal performance curves can be applied to microscopic organisms, including pathogens (Ratkowsky et al., 1983; Verant et al., 2012; Voyles et al., 2017). Thus, TPCs are often used in disease studies to quantify how environmental temperatures may regulate disease dynamics. Temperatures can influence both pathogens and hosts and their relationship (Cohen et al., 2017; Gehman et al., 2018; Kirk et al., 2005; Linder et al., 2008). Pathogenic characteristics, such as their distributions, growth rates, and survival rates, can be affected by environmental temperatures (Harvell et al., 2002; Lafferty, 2009; Stevenson et al., 2013).

The effects of temperature on disease have often been studied in controlled constant temperature environments (Adamo & Lovett, 2011; Stevenson et al., 2013; Verant et al., 2012; Voyles et al., 2017). These data can be used to predict how an organism will perform in more natural thermal conditions because experiments using more complex, fluctuating temperatures are often rare (Greenspan et al., 2017). However, extrapolating from these constant temperature experiments and TPCs to varying temperature regimes (as are experienced in almost all natural thermal conditions) is difficult and the accuracy of doing so is still debated (Bernhardt et al., 2018; Liu et al., 1995; Niehaus et al., 2012). It is important to improve methods to generalize from constant temperature experiments to more natural varying temperature environments due to the numerous host and pathogen characteristics that temperature can influence (Adamo & Lovett, 2011; Bailey et al., 2017; Stevenson et al., 2013; Ward et al., 2007).

The amphibian chytrid fungus, *Batrachochytrium dendrobatidis* (*Bd*), is a fungal pathogen that causes chytridiomycosis (Berger et al., 1998; Fisher et al., 2009). This disease has been linked to amphibian population declines worldwide (Scheele et al., 2019). Field studies have found that *Bd* infections are strongly affected by temperatures. For example, infection prevalence and intensity tend to be lower in warmer seasons, lower elevations, and in warmer aquatic and terrestrial habitats (Berger et al., 2004; Forrest & Schlaepfer, 2011; Roznik et al., 2015). Laboratory studies have shown that *Bd* growth and survival are limited at temperatures over 30°C and optimal growth occurs at temperatures between 17 and 25°C (Piotrowski et al., 2004; Stevenson et al., 2013; Woodhams et al., 2008). *Bd* can grow at temperatures as low as 4°C, although growth at this temperature is slow (Piotrowski et al., 2004; Voyles et al., 2012). Recently, research has also shown how temperature fluctuations can impact *Bd*'s growth rate (Lindauer et al., 2020; Stevenson et al., 2020). Fluctuating temperatures and heat pulses can reduce mortality and morbidity in amphibian hosts (Greenspan et al., 2017; Raffel et al., 2015; Woodhams et al., 2003).

In this study, we assessed the capability of a hierarchical model fit to constant temperature data to predict measurements taken under known fluctuating temperature regimes, using *Bd* grown in vitro as our study system. We used data collected by Stevenson et al. (2013) and Stevenson et al. (2020) and previously modeled in a different manner by Greenspan et al. (2017). Specifically, we used *Bd* optical density growth data collected at 10 constant temperature data by Stevenson et al. (2013) to fit a hierarchical logistic growth model. In this model, we constrained the logistic growth rate by a TPC. To test how the choice in TPC influenced the model, we fit the model five times with the same constant temperature data and altered the TPC used in the model. We then used these five fitted models to make predictions about growth in three varying temperature scenarios. We then validated these predictions by using *Bd* data grown in the three varying temperature scenarios.

Given previous work (Kingsolver & Woods, 2016; Ma et al., 2015; Tomanek, 2010), we expected that our simple method of generalizing from constant to time‐varying temperatures would perform least well for varying temperatures near the TPC peak, due to the non‐linearity. We also expected that the generalization would perform best under the varying temperature regime with the smallest daily fluctuations. Lastly, we expected that the TPCs that had a typical left‐skewed shape would make more accurate predictions due to their common use and model comparisons in the literature (Huey & Kingsolver, 1989; Rebaudo et al., 2018; Shi & Ge, 2010).

## 2 METHODS

### 2.1 *Bd* cultures

The *Bd* strain Paluma‐Lgenimaculata #2‐2011‐CO was used for both constant and varying temperature trials in this study. This strain was isolated from a green‐eyed tree frog tadpole (*Litoria serrata*) near Paluma, Queensland, Australia, using the protocol described in Stevenson et al. (2013). The strain was cultured in TGhL broth (8 g tryptone, 1 g gelatin hydrolysate, and 2 g lactose in 1 L of distilled water) and passaged 24 times before the constant temperature experiments and 12 times for the varying temperature experiments.

### 2.2 Constant temperature experiments

Here, we briefly review the protocols used by Stevenson et al. (2013) to collect constant temperature optical density growth measurements. *Bd* was grown on TGhL agar plates prior to the study. They allowed the *Bd* to grow for three days, on these plates, before flooding the plates with TGhL broth. The TGhL broth used to flood the plates was recollected and filtered to remove zoosporangia. Zoospores in the filtered solution were quantified with a hemocytometer. The zoospore concentration in the filtered solution was found to be $5.75\times 10^{7}$ zoospores per ml. The zoospores solution was then used to inoculate 96‐well plates used in the constant temperature growth experiments.

Each 96‐well plate consisted of 18 positive wells (100 μl of the filtered zoospore solution), six negative wells (100 μl of heat‐killed *Bd*, 60°C for 45 min), and 24 wells containing 100 μl of TGhL. The plates were randomly assigned to 1 of 10 constant temperature treatment (13, 15, 17, 19, 21, 23, 25, 26, 27, and 28°C).

For each of the plates, Stevenson et al. (2013) measured optical density of *Bd* growth in each well every day from day 0 to day 14. Optical density was measured using a Multiskan Ascent 96/384 Plate Reader (MTX Lab Systems Incorporated) at 492 nm. Plates were checked daily for contamination, usually seen by high optical density readings and/or discoloration in the well. Contaminated wells were not included in the final analysis. All optical density readings were adjusted by subtracting out the mean of the negative control wells (heat‐killed *Bd* wells).

### 2.3 *Bd* optical density logistic growth model

**FIGURE 1:**
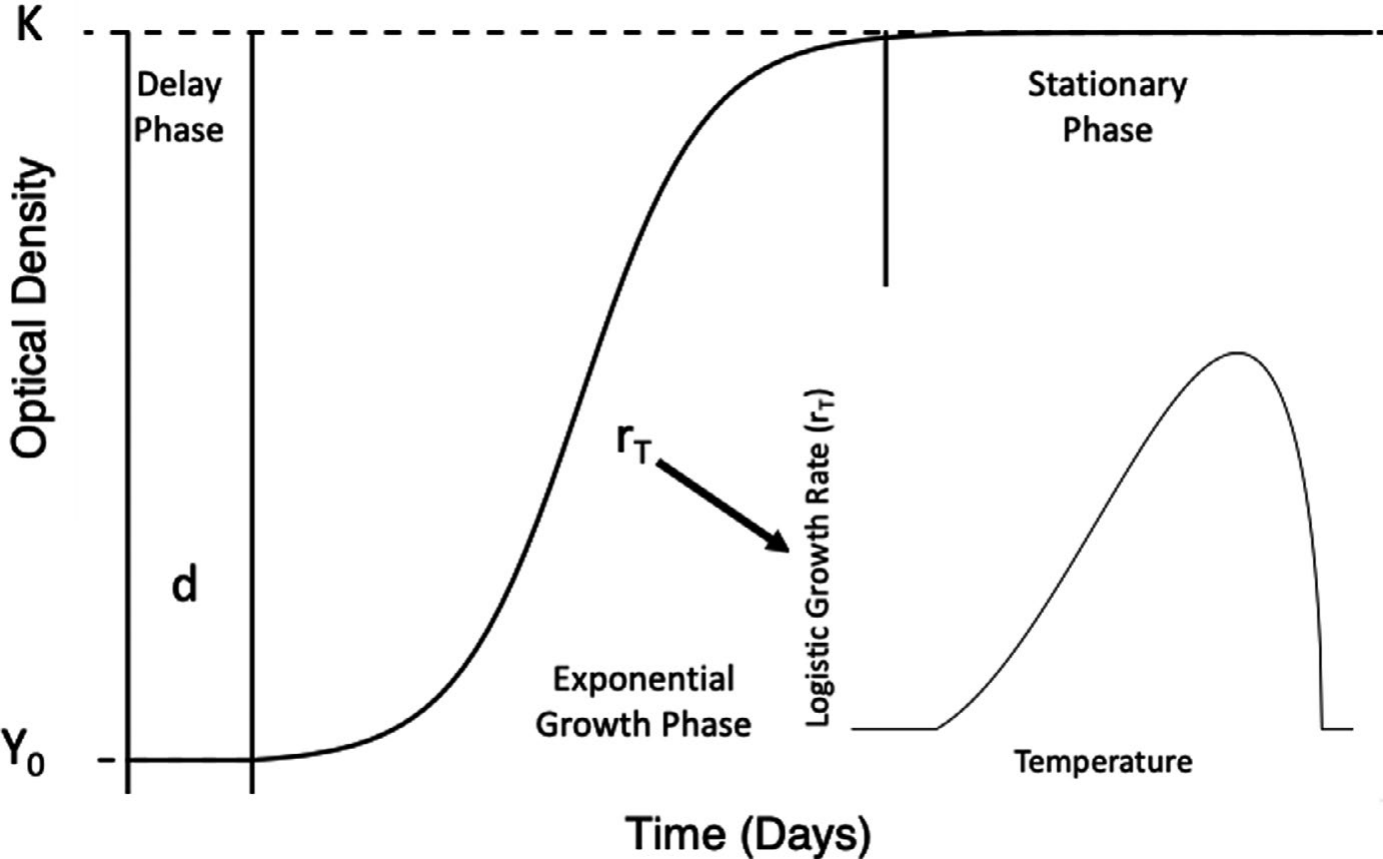
Figure shows a graphical representation of Equation (1) used to fit the *Bd* optical density data and also shows the three phases of the model. The model starts out at time 0 and $Y_{0}$ and enters the delay phase. The length of the delay phase is controlled by d. The steepness of the exponential growth phase is controlled by $r_{T}$ and the stationary phase is reached once the optical density value is equal to K. The logistic growth rate, $r_{T}$, is temperature sensitive is regulated by a thermal performance curve (shown in the bottom right corner)

We assumed that the *Bd* optical density growth pattern could be described by a logistic growth model that had a delay period before *Bd*’s exponential growth (Figure 1). Specifically, we used the following equation:

(1) $$Y\left(T_{i}\right)=Y_{0}1_{\left(t_{i}<d\right)}+\frac{KY_{0}}{Y_{0}+\left(K-Y_{0}\right)e^{-r_{T}\left(t_{i}-d\right)}}1_{\left(t_{i}\geq d\right)}$$

to fit *Bd* optical density growth patterns, where $Y(t_{i})$ is the initial optical density measurement, d is the delay period, K is the maximum optical density, $Y_{0}$ is the initial optical density, $r_{T}$ is the logistic growth rate, and $t_{i}$ is the ith time. This logistic growth model was first described in Voyles et al. (2017) and used to fit *Bd* optical growth patterns (Figure 1). The logistic growth equation (Equation 1) was modified to add a delay period due to the time it takes zoospores to settle in the well and develop into zoosporangia. This phase is known as the delay phase and lasts for d amount of time. Next, wells enter the exponential phase where zoosporangia release new zoospores and those new zoospores settle and develop into zoosporangia. The rate at which this happens is controlled by $r_{T}$; the higher the value of $r_{T}$, the faster the growth and development of *Bd*. Lastly, there is the stationary phase when optical density is not increasing anymore due to new zoosporangia not releasing new zoospores into the wells.

### 2.4 Thermal performance curve

We found several thermal performance curves (TPCs) in the literature. The TPCs found varied in functional form and were developed for a wide range of organisms. Most of the TPCs found were phenomenological models, but some were mechanistic models based on enzyme kinetics. We chose five TPCs from the literature that varied in function and purpose for development. The models we selected were as follows: Briere 2 (Briere et al., 1999), Ratkowsky (Ratkowsky et al., 1983), Ikemoto (Ikemoto, 2005), Logan 10 (Logan et al., 1976), and Stinner (Stinner et al., 1974). The TPCs selected were used to explore how different TPC shapes and mathematical functions could affect predictions made about *Bd* growth in a varying temperature environment. All TPCs chosen had the logistic growth rate ( $r_{T}$) go to zero at temperatures below $T_{\min}$ and above $T_{\max}$. This choice was made because *Bd* growth was measured with optical density, which measures everything in the sample, including both live and dead cells. Therefore, we do not expect *Bd* optical density measures to decrease. All parameter definitions for the chosen TPCs can be found in Appendix S1.

The five TPCs chosen ranged in the number of parameters (4–8). The five functions also varied in whether they included critical thermal parameters, such as $T_{\min}$, $T_{\mathrm{opt}}$, and $T_{\max}$. Briere 2, Logan 10, and Ratkowsky each have two critical thermal parameters while Ikemoto and Stinner each have only one (Briere et al., 1999; Ikemoto, 2005; Logan et al., 1976; Ratkowsky et al., 1983; Stinner et al., 1974). Most functions came from the entomology literature, with Logan 10 being one of the most popular. Ratkowsky is an outlier in the TPCs we consider, coming from the microbial literature. The Stinner model, the oldest model that we included, has a distinct tabletop shape that differed from the other TPCs. Lastly, some of the TPCs chosen do not have true $T_{\min}$ or $T_{\max}$ values because they asymptotically approach zero. Thus, for these functions, the logistic growth rate ( $r_{T}$) will never be exactly zero. In order to allow comparisons between all models, we define effective $T_{\min}$ and $T_{\max}$ for each TPC, which are the lower and upper temperatures where the logistic growth rate (defined by the TPC) becomes ≤0.01.

### 2.5 Fitting the logistic growth model

We fit Equation (1) to the constant temperature *Bd* optical density growth data using a hierarchical Bayesian approach. That is, we fit all the constant temperature optical density data simultaneously using one logistic growth model (Equation 1). However, instead of placing a simple prior on the logistic growth rate ( $r_{T}$), we constrained the shape of the growth rate to conform to one of the five TPC functional forms as presented in the previous section. Each of the five was fit in turn, and the predictions of the fitted models were compared. Mathematically, the hierarchical model is given by:

$$\begin{array}{cc} Y\left(T_{i}\right) & ∼\log N\left(\mu_{i},\tau_{i}\right)\\ \mu \left(T_{i}\right) & =Y_{0}1_{\left(t_{i}<d\right)}+\frac{KY_{0}}{Y_{0}+\left(K-Y_{0}\right)e^{-r_{T}\left(t_{i}-d\right)}}1_{\left(t_{i}\geq d\right)}\\ r_{T} & =f\left(T,\theta \right)\\ \theta & ∼g(ϴ)\\ \tau_{i} & =1/\sigma_{i}^{2}\\ \sigma & ∼\mathrm{Exp}\left(0.001\right)\\ Y_{0} & ∼\text{Uniform}\left(0.001,0.01\right)\\ d & ∼\mathrm{Exp}\left(2\right)\\ K & ∼\text{Uniform}\left(0.1,0.6\right) \end{array}$$

This is the general formulation of the model where f(T,θ) denotes the functional form of a chosen TPC with parameter set θ and the priors distributions of the parameters determining the shape of the TPC are denoted by *g*(ϴ). Logistic growth parameters priors were based on Voyles et al. (2017) priors. Priors for the parameters in each of the TPC were based on values found in previous publications, which are given in devRate (Rebaudo et al., 2018). TPC priors are shown in Appendix S1.

We chose to fit the model hierarchically instead of fitting 10 separate logistic growth models (1 per constant temperature treatment) and then using posterior distribution samples to fit the thermal performance curves. Fitting this model hierarchically allows the logistic growth parameters and thermal performance curve parameters to be fit together and influence each other. This also gives the model extra information about the expected value for the logistic growth rate ( $r_{T}$). For example, fitting a logistic growth model to the 28°C treatment where there is little to no *Bd* growth is difficult. This is because there are three different ways in the model for this pattern to be achieved and have a long delay period ( d), a low maximum optical density ( K), or a low logistic growth rate ( r). The hierarchical modeling approach provides the logistic model with information about what the expected logistic growth rate ( $r_{T}$) should be. The extra information on $r_{T}$ then influences the estimates of d and K, allowing for a better fit to this temperature treatment.

Parameters from the model were estimated via MCMC using the rjags package (Plummer, 2003) in R (Team & R. C., 2013). For each fitted model, five chains were run for 20,000 iterations and the first 10,000 samples discarded for burn‐in for a total of 50,000 MCMC samples from the posterior distribution for each model. Trace plots of the MCMC chains were checked visually for convergence. We also checked chains for autocorrelation. Two of the hierarchical models exhibited high autocorrelation. In these cases, the number of iterations was increased from 10,000 to 100,000 then thinned (keeping every 100th sample) to maintain the same number of posterior samples.

To compare the different hierarchical model fits to the constant temperature optical density data, we used deviance information criterion (DIC), which is defined by:

(2) $$\text{DIC}=\bar{D}+pD$$

where $\bar{D}$ is the expected deviance and is added to (*pD*), effective number of parameters (Spiegelhalter et al., 2002). The *pD* was defined as the one proposed by (Plummer, 2002). We used DIC for model comparison due to its use for Bayesian model comparison by taking into account the fit (expected deviance) and complexity (effective number of parameters) of the model (Spiegelhalter et al., 2002). The DIC calculations for each hierarchical model were done in rjags using the dic.sample function (Plummer, 2003). The DIC values were compared, and the model with the lowest DIC value was considered the best fit to the constant temperature data (Table 1).

**TABLE 1:** Adjusted $T_{\min}$, adjusted $T_{\max}$, maximum $r_{T}$, penalized deviance values, and ΔDIC, for each thermal performance curve used in the hierarchical models

TPC function	Adjusted $T_{\min}$	Adjusted $T_{\max}$	Maximum $r_{T}$	Pen. dev.	ΔDIC
Stinner	10.95 (10.86, 11.04)	27.52 (27.44, 27.58)	0.813 (0.789, 0.839)	−31,269	0
Logan 10	−0.03 (−0.15, 0.06)	26.98 (26.98, 26.98)	0.852 (0.826, 0.881)	−30,369	900
Briere 2	7.33 (6.01, 8.39)	27.92 (27.78, 27.99)	1.070 (1.015, 1.128)	−28,076	3193
Ratkowsky	3.07 (1.45, 4.60)	28.31 (28.22, 28.40)	1.166 (1.106, 1.238)	−28,055	3214
Ikemoto	2.93 (2.64, 3.18)	40.35 (40.30, 40.42)	1.010 (0.991, 1.021)	−28,014	3255

Notes

The $T_{\min}$, $T_{\max}$, and $r_{T}$ columns show the posterior mean and the 95% highest posterior density interval in parentheses. Adjusted $T_{\min}$ and $T_{\max}$ are defined at the temperatures at which the posterior medians of the logistic growth rate reach 0.01. Maximum $r_{T}$ is the temperature at which the logistic growth rate is maximized. Penalized deviance column shows the values calculated by rjags using the dic.sample function. Lastly, the ΔDIC is defined by $\Delta \mathrm{DIC}={\mathrm{DIC}}_{i}-{\mathrm{DIC}}_{\min}$. The DIC indicates that the Stinner model is the best fit to the constant temperature data.

### 2.6 Varying temperature experiments

**FIGURE 2:**
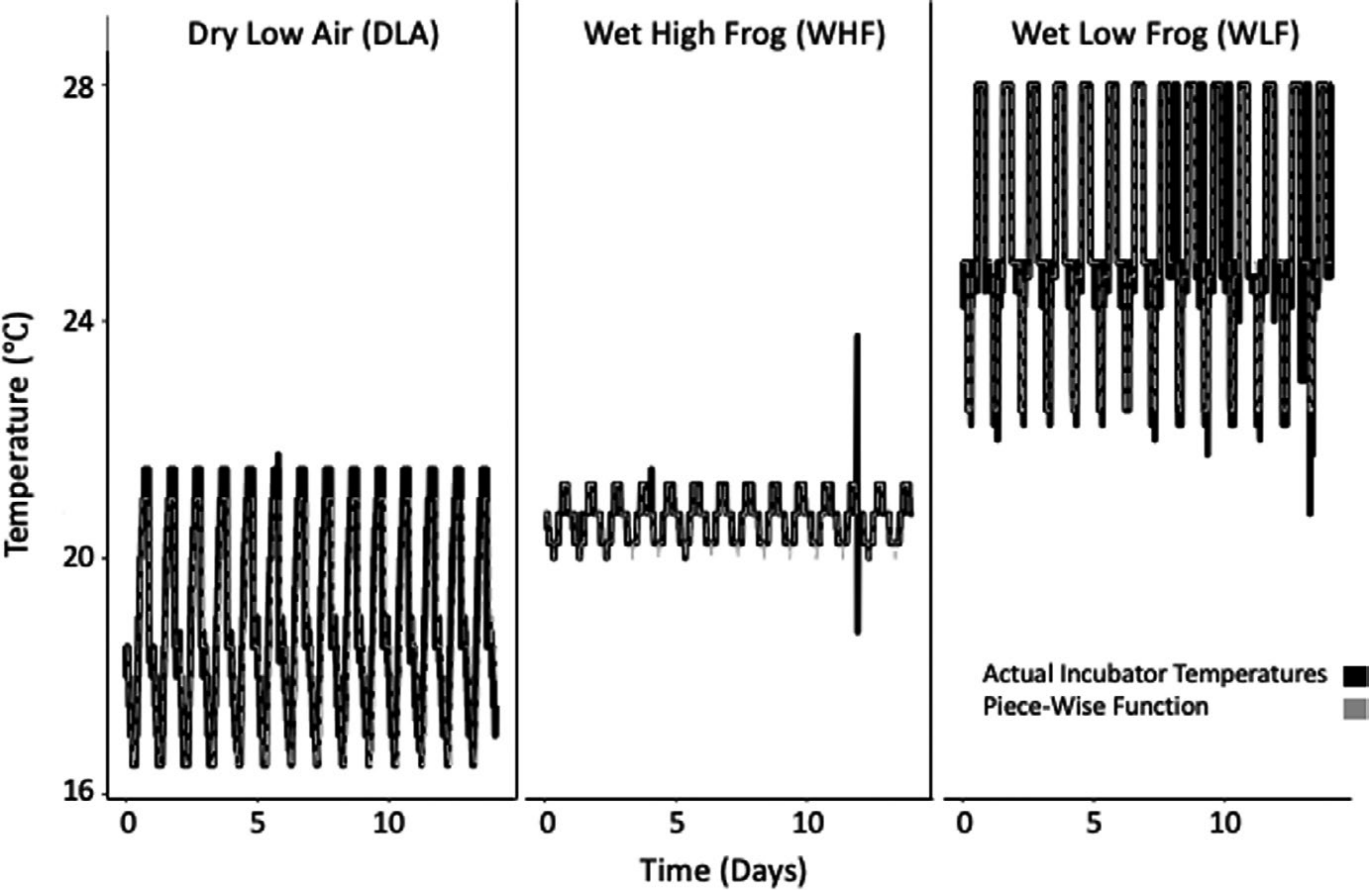
Plotted are the varying temperature regimes that were used to grow *Bd* in incubators. The three regimes are based on temperatures recorded in either low or high altitude, wet or dry seasons, and air or on a frog. Black lines are temperatures recorded in the incubator over time, while the dashed gray lines represent the piece‐wise functions fit to the incubator temperatures. There were some inconsistencies in the thermal data, as seen in the spike in the wet high frog regime and when the wet low frog cycle went out of sync. The dry low air also had tiny spikes that the piece‐wise function did not take into account due to the short time frame of the spikes

We validated our predictions made from the constant temperature hierarchical models with varying temperature *Bd* optical density data (Stevenson et al., 2020). The varying temperature regimes were realistic simulations of regimes experienced by amphibians based on temperature loggers placed in the environment. Thermal regimes were recorded during the wet summer season and dry winter season, at low and high elevations, and in the water, air, or in a frog model (made from agar, using models with both perfect and zero resistance to evaporative water loss; Roznik & Alford, 2014, 2015). The agar frog models were placed in daytime and nighttime locations used by tracked frogs (Roznik & Alford, 2015). The incubator temperature regimes were created by taking the logged temperature information and averaging it (separately for daytime and nighttime locations) and splitting it into 4‐h intervals. A temperature logger was placed in each incubator to record the temperature during the experiments (Figure 2).

We only used data from 3 out of the 12 varying temperature regimes (all reported in Stevenson et al. (2020)) because of natural weather fluctuations in the other nine datasets made it difficult to evaluate consistency in thermal regimes across days. This constraint highlights that our method and other methods using integration (Worner, 1992) are essentially unable to evaluate 75% of our data, which was collected under natural weather conditions. We used three varying temperature regimes (shown in Figure 2) in our analyses because they had the most consistent temperature fluctuations over time (across days), whereas the other temperature regimes varied much more widely and unpredictably in their fluctuations over time, which is representative of the weather conditions and thermoregulatory behavior of the animals studied at that time (Figure 2).

The experimental setup for the varying temperature 96‐well plates was the same as for the constant temperature experiments. The same *Bd* strain and *Bd* starting concentration were used in the varying temperature experiments as the constant temperature experiments, the only differences were the *Bd* was passaged an additional 12 times before the varying temperature experiments. This was due to the varying temperature experiments being conducted 2 months after the constant temperature experiments. After the 96‐well plates were inoculated, they were placed in varying temperature incubators that used one of three varying temperature treatments (Dry Low Air, Wet High Frog, or Wet Low Frog). Optical density *Bd* growth measurements were taken daily, for each well and plate, for 12 days using the same protocol as the constant temperature treatments.

### 2.7 Predicting *Bd* performance in varying temperature regimes

To make predictions about how *Bd* grew in the three varying temperature regimes, we used the following logistic growth function:

(3) $$\frac{dx}{dt}=r_{T}x\left(\frac{1-x}{K}\right){\mathrm{II}}_{\left(t>d\right)}$$

where x is optical density, $r_{T}$ is the temperature‐dependent logistic growth rate, and K is the maximum optical density. With this model, we were able to integrate over time and use a time‐varying temperature regime to inform the logistic growth rate ( $r_{t}$) value. The logistic growth rate ( $r_{t}$) was solved for from one of the five TPCs. To determine what the time‐varying temperature should be at each time point, we fit three piece‐wise functions, one to each of the varying temperature regimes, and used these to solve for the temperature at time i. Parameters for both the thermal performance curve and logistic growth model were from 1000 samples taken from the posterior distribution of each hierarchical model (from the fits utilizing each of the 5 TPCs).

We then integrated the logistic growth model (Equation 3) over 16 days by intervals of 0.001 to predict *Bd*’s growth in each of the varying thermal regimes for each of the five hierarchical models. Integration was done using deSolve and the default lsoda integrator (Soetaert et al., 2010). Time was linked to temperature by the piece‐wise function fit to the incubator temperature data (Figure 2). We made 1000 predictions, based on the 1000 parameter values sampled from the posterior distributions. We calculated the median prediction and the 95% highest posterior density interval for both the optical density growth prediction and the TPCs.

We wanted to compare predictions about the parameters in the logistic growth model so we refit Equation (1), once per varying temperature regime (*n* = 3), without constraining the logistic growth rate by a TPC. This model was fit with rjags and used similar priors to the hierarchical model and $r_{T}$ had a prior this time (Gamma (1,1)). We also assigned $Y_{i}$ a normal distribution due to negative optical density values in the varying temperature treatments. We ran the model for the same number of iterations and removed the same burn‐in. We then compared our predicted parameter values to these parameter values, found by just fitting a non‐temperature‐dependent logistic growth model.

## 3 RESULTS

### 3.1 Thermal performance curve models

**FIGURE 3:**
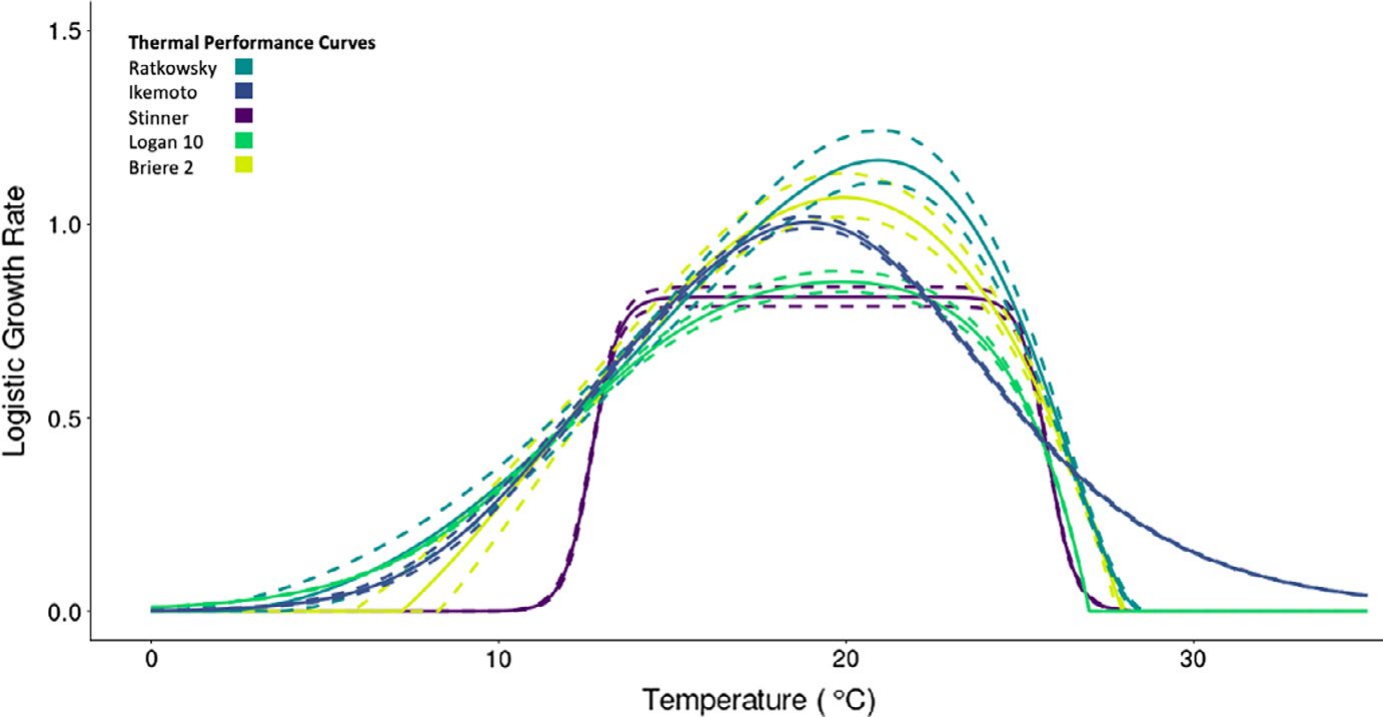
Thermal performance curves were created from 1000 samples from the posterior distribution of each of the hierarchical models. The output from five thermal performance curves used to constrain the logistic growth rate. Dashed lines represent 95% credible intervals and solid lines represent medians

The relationships between the logistic growth rate ( $r_{T}$) and temperature in the hierarchical models were constrained by the thermal performance curves (TPC) used. The structure of the Briere 2, Logan 10, and Ratkowsky models results in similar relationships between *Bd* growth rate and temperature—that is, these three models have a left‐skewed shape. In contrast, the Ikemoto model is more symmetrical (Figure 3). The Stinner model is also symmetrical, but is nearly constant at intermediate temperatures.

We compare predictions of key thermal parameters such as $T_{\min}$, $T_{\mathrm{opt}}$, and $T_{\max}$ which differed due to using different TPCs to constrain the shape of the logistic growth rate over temperature. At lower temperatures, the predicted logistic growth rates ( $r_{T}$) patterns were similar in the Logan 10, Ikemoto, and Ratkowsky models. These three models have a less drastic reduction in $r_{T}$ as temperature decreases compared to the other two models. The Briere 2 and Stinner models had higher adjusted $T_{\min}$ and a more distinctive decrease in $r_{T}$ as temperatures become cooler. The Stinner model had the highest median adjusted $T_{\min}$ at 10.95°C (Table 1). There were similar patterns at the upper end of the TPCs. All models except the Ikemoto model predicted a sharp drop in $r_{T}$ at temperatures over the predicted thermal optimums (Figure 3). The Ikemoto model also had a much higher $T_{\max}$ than the other four models (Table 1).

Hierarchical models assuming the Logan 10 and Briere 2 TPCs were the only ones that predicted similar temperatures for their thermal optimums, with median values of 19.86 and 19.92°C, respectively (Table 2). The lowest optimal temperature predicted was 18.92°C from the Logan 10 model, while the highest optimal temperature was 20.94°C predicted from the Rakowsky model. At the TPCs’ optimal temperatures, we found that the predicted maximal logistic growth rate for each TPC varied considerably. The Ratkowsky and Briere 2 models had the two highest maximal logistic growth rates ( $r_{T}$). The Ikemoto had the third highest $r_{T}$ and was not significantly different than the Briere 2 $r_{T}$value (Table 1). The last two models, Stinner and Logan 10, had significantly lower (but similar) maximal $r_{T}$ values compared to the other three models.

**TABLE 2:** Comparison of estimated logistic model parameters (excluding r(T)) obtained under the 5 assumed thermal performance curve functions

Param	Briere 2	Ratkowsky	Ikemoto	Logan 10	Stinner
d	1.026 (0.985, 1.114)	1.065 (0.993, 1.186)	0.007 (0.001, 0.033)	0.220 (0.055, 0.381)	0.356 (0.191, 0.539)
K	0.122 (0.113, 0.131)	0.119 (0.110, 0.128)	0.135 (0.133, 0.136)	0.134 (0.133, 0.136)	0.134 (0.133, 0.136)
$T_{\min}$	7.214 (5.949, 8.403)	274.6 (272.9, 276.5)	NA	NA	NA
$T_{\mathrm{opt}}$	19.92 (19.74, 20.11)	20.94 (20.68, 21.19)	18.92 (18.87, 18.97)	19.86 (19.80, 19.93)	19.23 (19.22, 19.25)
$T_{\max}$	27.95 (27.806, 28.040)	301.6 (301.4, 301.7)	NA	27.001 (27.000, 27.005)	NA

Notes

For each model, we report the posterior median values and 95% highest posterior density intervals values calculated from samples from the posterior distributions (*N* = 1000). Medians and highest posterior density intervals are also shown for the critical thermal values ( $T_{\min}$, $T_{\mathrm{opt}}$, and $T_{\max}$) if these were estimated parameters in the given model (adjusted $T_{\min}$ and $T_{\max}$ are shown in the supplemental material. All median values for $Y_{0}$ are $1\times 10^{-3}$ and have all models have an interval that falls between $\left(1\times 10^{-3},1.04\times 10^{-3}\right)$.

Given the differences in patterns caused by the various TPCs, we sought to evaluate which of these was most consistent with the data used for fitting. We compared the deviance information criteria (DICs) from the five hierarchical model fits to the constant temperature data. Surprisingly, the Stinner model has the lowest DIC value, indicating that this model fit the constant temperature optical density data the best (Table 1). The Logan 10 model was the second best fit having a ΔDIC value of 900 (Table 1), indicating that even this second model performed significantly more poorly fitting these data. The other models performed even more poorly. This indicates that the models with similar intermediate temperature logistic growth rates and lower maximal logistic growth rates did a better job of fitting the constant temperature data.

### 3.2 Hierarchical model parameters

Along with the TPC parameters, the hierarchical models also had differences in the logistic growth model parameters, specifically the carrying capacity, K, and the length of the delay period, d. The delay parameter ranged from 1.065 in the Ratkowsky model to 0.007 in the Ikemoto model. The Briere 2 model had a similar delay to the Ratkowsky model, while the Logan 10 and Stinner d values were similar. The five different maximum optical density parameter values, K, fell into two significantly different groups. Briere 2 and Ratkowsky had lower K values predicted, while the Ikemoto, Logan 10, and Stinner models predicted a significantly higher K value (Table 2). In contrast, the estimated initial optical density, $Y_{0}$, was the same across all models.

### 3.3 Predicting optical density under varying temperature conditions

**FIGURE 4:**
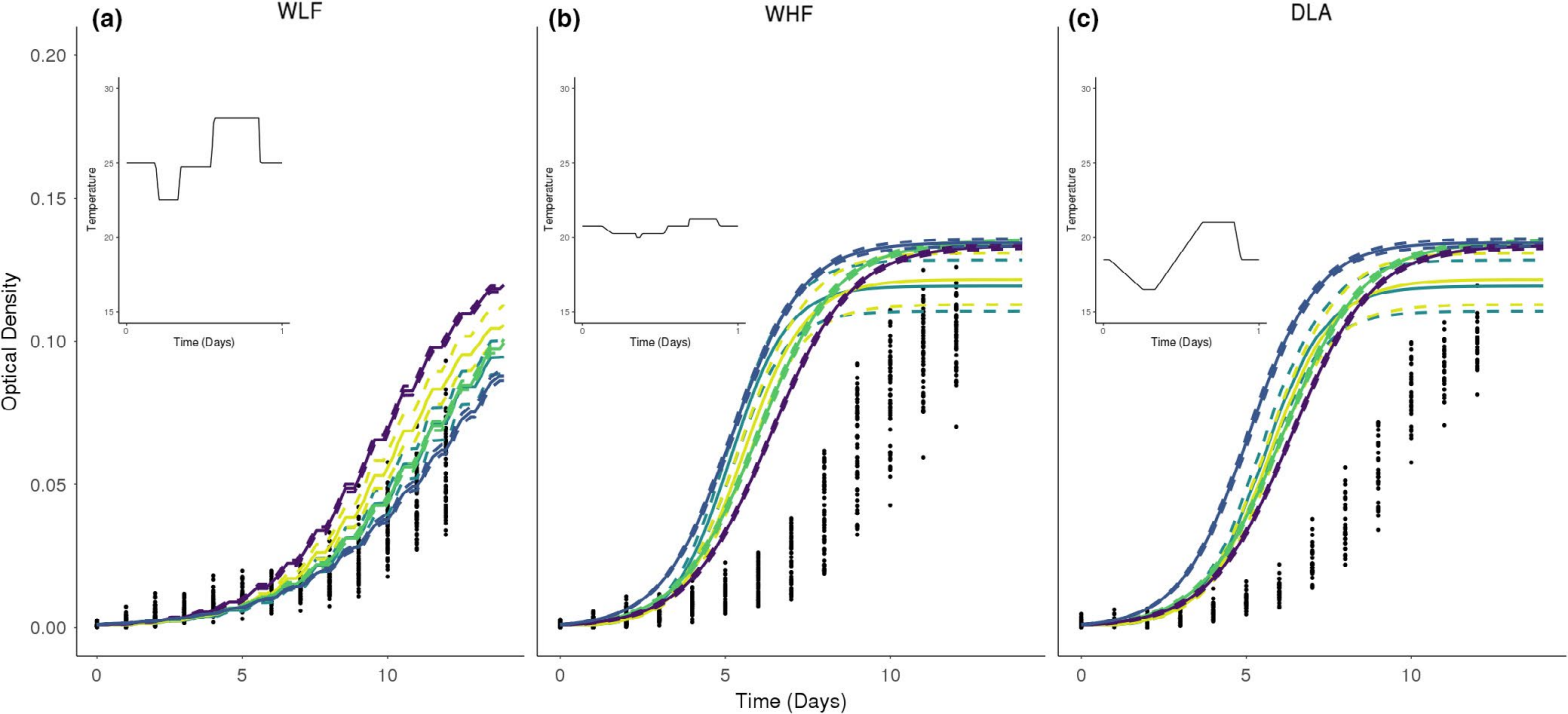
Varying temperature predictions at three different temperature regimes; (a) Wet Low Frog (WLF), (b) Wet High Frog (WHF), and (c) Dry Low Air (DLA). Each panel shows predictions made from five different thermal hierarchical models. Dashed lines represent the 95% credible interval, while the solid lines represent median predictions from 1000 samples. The points in each panel represent optical density measurements taken under the specified temperature regimes. Above each prediction plot shows what the corresponding fluctuating temperature regime looks like for 24 h

We attempted to use the fitted models from the previous sections to predict optical density measurements under three time‐varying temperature regimes (predictions shown in Figure 4) and compared their performance to a simple logistic model fit directly to the time‐varying data. All models overpredicted optical density growth at the low and intermediate, Dry Low Air (DLA) and Wet High Frog (WHF), temperature regimes. However, the Ikemoto model, which performed the worst at predicting optical density growth in the DLA and WHF temperature regimes, did the best at predicting growth at the highest temperature regime, while the Stinner model, which was the closest to accurately predicting optical density growth in the DLA and WHF temperature regimes, made the worst prediction at the highest temperature regime, Wet Low Frog (WLF).

The WHF regime exhibits the least amount of variation between maximum and minimum temperatures. Again, the Stinner model performed the best and had a closer average logistic growth rate to the logistic growth rate predicted at that temperature regime (see Appendix S1 for values). The Ikemoto, again, was the worst at predicting *Bd* growth at the WHF temperature regime. However, at the WHF temperature regime, the Ikemoto model made a better prediction compared to the DLA prediction and was closer to predictions made by the other four models. The other models, Briere 2, Ratkowsky, and Logan 10, were all similar but overlapped less with each other.

Lastly, the WLF temperature regime had the highest mean and maximum temperature, with a range that spans most of the thermal maxima estimated by the hierarchical models. The Stinner model, which had the best *Bd* growth predictions in the other two varying temperatures, made the worst prediction at this temperature regime. The Ikemoto model, which had the worst predictions in the other two temperature regimes, made the best prediction in the WLF temperature regime. The Logan 10 and Ratkowsky models, which made similar predictions, did overpredict *Bd* growth but not by a significant amount. Briere 2 again overpredicted *Bd* growth at this temperature regime but made a more accurate prediction at this temperature regime when compared to the WHF and DLA temperature regime predictions.

## 4 DISCUSSION

In this paper, we explored the extent to which TPCs estimated under multiple constant temperatures can be generalized to make predictions of growth of a fungal pathogen, *Bd*, under varying temperature regimes. We also explored how changing the assumed form of the TPC used to constrain the logistic growth rate ( $r_{T}$) in the hierarchical model affected predictions. Surprisingly, we found that our method did not make the most accurate predictions with the temperature regime that had the smallest fluctuations or with temperature regimes with more intermediate temperatures. We also found that which TPCs were used in the hierarchical model made a difference and that the more typical left‐skewed TPCs, like Briere 2, did not make the best predictions. This reinforces the inadequacy of current methods for predicting thermal performance under time‐varying temperature conditions, even for these small organisms, from measurements taken at constant temperatures.

All the hierarchical models generally overpredicted the growth of *Bd* in the three different varying temperature regimes. However, which ones performed best or worst were often surprising. For example, the Ikemoto model (with the highest thermal maximum) made the most accurate prediction for the warmest varying temperature regime (WLF), but the worst predictions for the other two varying temperature regimes. The Stinner model was the reverse, performing the best for the intermediate and lower temperature regimes but making the worst prediction for the highest temperature. By examining these best and worst fitting models, we may be able to parse out what features of the TPCs are improving or hampering predictions. For example, the Ikemoto curve does not have a drastic drop off in growth rate after the thermal optimum, which may have allowed improved prediction in the high temperature regime. Another example is the Stinner model's predictions at intermediate temperatures, which were predicted to all be very similar. These factors and how the TPCs influence the logistic growth parameters, such as the delay rate ( d) (which was lower than expected in the Ikemoto model), highlight the need to take the function and shape of the TPC into account and consider what they might say about the biology of the trait.

Some predictions that our hierarchical models made about *Bd* optical density growth are inconsistent with the known biology of *Bd*. The fitted Stinner model predicts little to no growth at lower temperatures ( <10°C), while the Logan 10 model predicted growth at close to 0°C. The Ikemoto model predicted *Bd* to continue growing at temperature >40°C. Based on other laboratory studies, we know that the *Bd* does not grow at temperatures >40°C and can grow at temperatures <10°C (Piotrowski et al., 2004; Stevenson et al., 2013; Voyles et al., 2017). Although both the Stinner model and Ikemoto model make better predictions in different varying temperature regimes than the other models, they make predictions that are not consistent with known *Bd* critical thermal values.

To improve model predictions, TPCs should be improved by incorporating more data into the model. For example, more data from constant temperatures below 13°C could shift the thermal minimum to more reasonable values and refine the shape of the TPCs at low and intermediate temperatures. Similarly, constant temperature data above 28°C, which would show little to no growth of *Bd*, could restrain TPCs predicting thermal maximums above this temperature. This could remove error due to extrapolating into temperatures for which we do not have data. This emphasizes the need to collect data over a range of temperatures to provide enough information to the model to make accurate predictions. Lastly, another way to improve model predictions is to ensure that constant temperature and varying temperature data are collected in the same time frame. This is due to some organisms, such as *Bd*, being able to adapt to laboratory conditions (Voyles et al., 2012). Adapting to laboratory conditions could alter an *Bd*’s performance in different thermal environments resulting in added error in *Bd* growth predictions. Although constant and varying temperature data used in this study were collected at different times (two months apart, or 12 passages from the original culture), we believe that not enough time had passed between the two data collection times to allow for significant *Bd* adaptation to laboratory conditions. Our model significantly over‐predicted *Bd* growth and a slight improvement from data collected at the same time would still lead to our model over predicting *Bd* growth.

The impact of temperature variability on the performance of organisms is being explored in the literature, and more studies are trying to predict performance in varying environments (Bernhardt et al., 2018; Denny, 2019; Ferguson & Sinclair, 2020; Khelifa et al., 2019; Kingsolver et al., 2015; Niehaus et al., 2012). The accuracy of methods, such as rate summation, which integrates over time‐varying temperature to determine growth or trait performance at a given time, is still under debate (Bernhardt et al., 2018; Liu et al., 1995; Niehaus et al., 2012). Most of these studies, similar to ours, deal with regular fluctuating temperature regime and do not make predictions on more irregular temperature regimes that might be caused by a weather event or thermoregulatory behavior. Current methods that integrate over time‐varying temperature regimes make it difficult to make predictions over irregular temperature regimes and test the accuracy of current methods. Integrating across time‐varying temperature requires temperature needed at every time point. We used a piece‐wise function on three repetitive temperature regimes in this study for this reason. However, more complex sporadic temperature regimes, like the nine temperature regimes in Stevenson et al. (2020) that were not used in this study, would require long piece‐wise function. A better understanding of the limitations of predicting varying temperature performance from constant temperature data and easier methods to make prediction on irregular temperature regimes are needed.

Thermal regimes are changing around the world (Easterling et al., 2000). Many publications (Chen et al., 2011; Hinder et al., 2014; Marshall et al., 2010; Narum et al., 2013) have suggested that if the thermal envelopes of areas shift beyond the tolerances of the organisms inhabiting them, those organisms will either die out, emigrate, or perhaps adapt. Changing temperatures could accentuate mismatches between host and pathogen performance curves and produce large changes in interactions (Cohen et al., 2017). However, this may underestimate the affects of the changing climate by not considering variation in temperature. Previous research has shown that temperature variation can affect species’ biology and interspecific interactions such as disease (Greenspan et al., 2017; Lindauer et al., 2020; Vasseur et al., 2014; Woodhams et al., 2003). However, for most species our understanding of the effects of temperature variation is poor and it is possible that changes in thermal regimes that do not overstep simple critical tolerances may still cause profound effects on species and species interactions, such as disease. Although some laboratory studies might help us determine how these temperatures might affect disease dynamics or organisms in certain locations, it would be time‐consuming and resource‐intensive to try to determine how an organism might perform in numerous varying temperature scenarios. However, if we improve our understanding of how an organism's performance in constant temperatures relates to their performance in varying temperatures, we would only need to measure a trait across several constant temperatures. This would require more constant temperature experiments with the pathogen and potentially a more holistic approach that examines both pathogen and host together. However, the current literature has a lot of constant temperate data (examples can be found in Adamo & Lovett[, 2011], Deutsch et al. [2008], Stevenson et al. [2013] and Voyles et al. [2017]) that can be used to refine techniques and guide further work.

We were unable to accurately predict an organism's performance in vitro in a varying temperature environment from constant temperature data, even using different TPCs and a hierarchical modeling approach that allows us to account for more uncertainty. This highlights a gap in our current models for understanding how organisms, even simple organisms in in vitro studies, react in varying temperature environments. Trying to make predictions with larger organisms we assumed that methods would need to be improved; however, we showed that even when trying to make predictions on smaller simple organisms, there is still a knowledge gap. Building off current methods, future studies could take some of these phenomenological functions and build more mechanistic functions explaining how an organism performance in varying temperature environments. With these models, we can start to determine what factors affect varying temperature performance and when current methods, like our hierarchical modeling approach, might be sufficient. For example, including an acclimation period for larger organisms might be needed instead of allowing the performance or a trait rate to immediately adjust based on the new temperature (Rohr et al., 2018). When temperatures exceed or drop below the thermal maximum and minimum, the organism could also accumulate damage and need to recover from that when temperatures are more optimal, further slowing the performance of a certain trait. Models will also have to make predictions with more irregular and sporadic temperature regimes, caused by weather events or thermoregulatory behavior. Building these new models will not only help improve our predictions of how organisms perform in varying temperature environments, but also improve our understanding of how temperature impacts organisms.

## AUTHOR CONTRIBUTIONS

**Zachary J. Gajewski:** Conceptualization (equal); data curation (equal); formal analysis (equal); funding acquisition (equal); investigation (equal); methodology (equal); project administration (equal); validation (equal); visualization (equal); writing—original draft (equal); writing—review & editing (equal). **Lisa A. Stevenson:** Data curation (equal); investigation (equal); methodology (equal); writing—review & editing (equal). **David A Pike:** Conceptualization (equal); data curation (equal); investigation (equal); methodology (equal); writing—review & editing (equal). **Elizabeth A Roznik:** Conceptualization (equal); data curation (equal); investigation (equal); methodology (equal); writing—review & editing (equal). **Ross Alford:** Data curation (equal); funding acquisition (equal); investigation (equal); writing—review & editing (equal). **Leah R Johnson:** Conceptualization (equal); formal analysis (equal); investigation (equal); methodology (equal); validation (equal); visualization (equal); writing—original draft (equal); writing—review & editing (equal).

## Supporting information

Appendix S1

## Data Availability

Constant temperature data used in this manuscript were from Stevenson et al. (2013) and varying temperature data were from Stevenson et al. (2020). Data and code for this manuscript will be publicly available on Zenodo at: https://doi.org/10.5281/zenodo.4525954.
