# Supplementary material for: Predicting the growth of the amphibian chytrid fungus in varying temperature environments
Source: Ecol Evol. 2021 Dec 17;11(24):17920–31. doi: 10.1002/ece3.8379 (PMC8717292; doi:10.1002/ece3.8379)
Supplement: Supplementary file 1 — Appendix S1 [file ECE3-11-17920-s001.pdf]

A Supplemental Figures

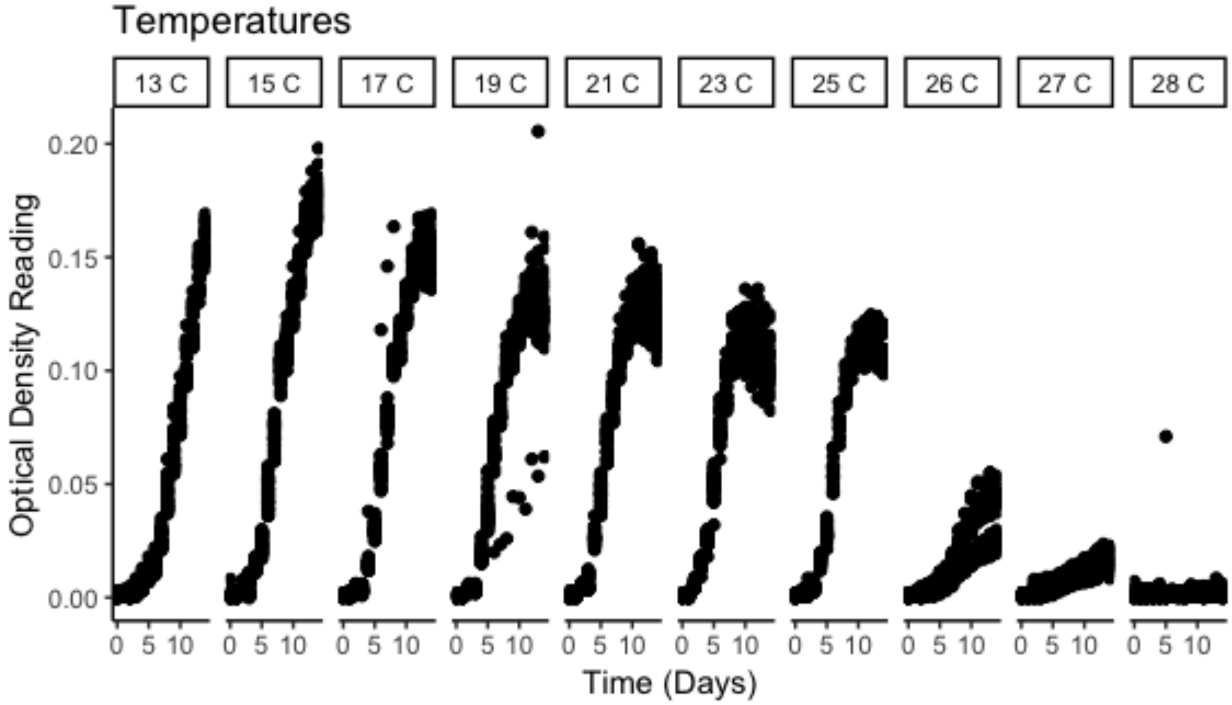

Figure A.1: Optical density readings at 10 different temperatures, for a North Queensland isolate of *Bd*, over 14 days.

B Supplemental Tables

Varying Temp Regime	r_T	K	d	Y_0
Dry Low Air	0.409<br>(0.388, 0.429)	0.196<br>(0.167, 0.227)	0.610<br>(0.132, 0.687)	0.0039<br>(0.0036, 0.0042)
Wet High Frog	0.443<br>(0.428, 0.460)	0.167<br>(0.154, 0.182)	0.156<br>(0.005, 0.339)	0.0035<br>(0.003, 0.0038)
Wet Low Frog	0.337<br>(0.321, 0.352)	0.125<br>(0.109, 0.146)	0.014<br>(0.00005, 0.058)	0.004<br>(0.004, 0.0041)

Table B.1: A logistic growth model (Eq. 1) was fit to each of the three varying temperature regimes. The median and 95% highest posterior density interval for each of the logistic growth models are shown in the table.

Temperatures	Ratkowsky	Briere 2	Ikemoto	Logan 10	Stinner
Dry Low Air	1.084<br>(0.926, 1.165)	1.044<br>(0.934, 1.069)	0.972<br>(0.916, 1.004)	0.841<br>(0.788, 0.851)	0.8126<br>(0.8126, 0.8129)
Wet High Frog	1.163<br>(1.159, 1.166)	1.060<br>(1.045, 1.068)	0.935<br>(0.896, 0.966)	0.847<br>(0.839, 0.851)	0.81269<br>(0.81269, 0.81270)
Wet Low Frog	0.719<br>(0, 1.101)	0.666<br>(0, 0.973)	0.494<br>(0, 0.773)	0.563<br>(0, 0.800)	0.720<br>(0.003, 0.813)

Table B.2: Based on the three temperature regimes, Dry Low Air (DLA), Wet High Frog (WHF), and Wet Low Fog (WLF), we calculated the median logistic growth rate that each thermal performance curve predicted. We also show the 95% highest posterior density interval, in parentheses, for each thermal performance curve.

Stinner<br>$r_T = \frac{c}{1+e^{(k_1+k_2T)}}(T_{opt} < T) + \frac{c}{1+e^{(k1+k2(2Topt-T))}}(T_{opt} > T)$			
Parameters	Definition	Prior Distribution	Posterior Median
c	Constant	$Gamma(1,1)$	0.813<br>(0.789, 0.839)
k_1	Constant	$N(32,1)T(30,40)$	32.364<br>(31.017, 33.983)
k_2	Constant	$N(0,5)T(-5,10)$	-2.555<br>(-2.450, -2.680)
T_{opt}	Thermal Optimum	$Uniform(13,25)$	19.234<br>(19.220, 19.250)

Table B.3: The table shows the Stinner thermal performance curve and all its parameters, the priors given to them, and the posterior median and highest posterior density intervals. All normal distributions are given as $N(\mu, \tau)$, where $\tau = \frac{1}{\sigma^2}$.

Briere 2<br>$r_T = cT(T - T_{min})(T_{max} - T)^{\frac{1}{b}}$			
Parameters	Definition	Prior Distribution	Posterior Median
c	Constant	$Gamma(1, 1)$	0.00049<br>(0.00044, 0.00055)
T_{min}	Minimum Temperature	$N(4, 0.5)$	7.214<br>(5.949, 8.403)
T_{max}	Maximum Temperature	$Uniform(27, 35)$	27.95<br>(27.806, 28.040)
b	Constant	$Gamma(10, 1)$	0.966<br>(0.895, 1.043)

Table B.4: The table shows the Briere 2 thermal performance curve and all its parameters, the priors given to them, and the posterior median and highest posterior density intervals. All normal distributions are given as $N(\mu, \tau)$, where $\tau = \frac{1}{\sigma^2}$.

Logan 10 $r_T = \alpha \frac{1}{1+ce^{-bT}} - e^{\frac{T_{max}-T}{\Delta_T}}$			
Parameters	Definition	Prior Distribution	Posterior Median
α	Constant	$Uniform(0, 2)$	0.134 (0.885, 0.957)
c	Constant	$N(90, 10)$	90.005 (89.409, 90.686)
b	Constant	$Gamma(1, 1)$	0.384 (0.378, 0.390)
Δ_T	Breadth of Range	$Gamma(1, 1)$	2.078 (1.995, 2.158)
T_{max}	Maximum Temperature	$Uniform(27, 35)$	27.001 (27.000, 27.005)

Table B.5: The table shows the Logan 10 thermal performance curve and all its parameters, the priors given to them, and the posterior median and highest posterior density intervals. All normal distributions are given as $N(\mu, \tau)$, where $\tau = \frac{1}{\sigma^2}$.

Ratkowsky83 $r_T = c(T - T_{min})(1 - e^{(k(T - T_{max}))})^2$			
Parameters	Definition	Prior Distribution	Posterior Median
c	Regression Coefficient	$Gamma(1,1)$	0.069 (0.059, 0.082)
k	Regression Coefficient	$Gamma(1,1)$	0.215 (0.181, 0.249)
T_{min}	Thermal Minimum	$N(277, .5)$	274.6 (272.9, 276.5)
T_{max}	Thermal Maximum	$Uniform(300, 308)$	301.6 (301.4, 301.7)

Table B.6: The table shows the Ratkowsky 83 thermal performance curve and all its parameters, the priors given to them, and the posterior median and highest posterior density intervals. All normal distributions are given as $N(\mu, \tau)$, where $\tau = \frac{1}{\sigma^2}$.

Ikemoto $r_T = \frac{\phi \frac{T}{T_\phi} e^{\frac{\Delta H_A}{R}(\frac{1}{T_\phi} - \frac{1}{T})}}{1 + e^{\frac{\Delta H_L}{R}(\frac{1}{T_L} - \frac{1}{T})} + e^{\frac{\Delta H_H}{R}(\frac{1}{T_H} - \frac{1}{T})}}$			
Parameters	Definition	Prior Distribution	Posterior Median
T_ϕ	Intrinsic Optimum Temperature	$Uniform(286, 298)$	291.5 (286.5, 295.3)
T_H	$\frac{1}{2}$ Enzymes Active at High Temperature	$Gamma(310, 1)$	294.4 (294.3, 294.5)
T_L	$\frac{1}{2}$ Enzymes Active at Low Temperature	$Gamma(273, 1)$	282.7 (282.3, 283.1)
ΔH_A	Enthalpy of activation	$N(16651, 5)$	16651.0 (16650.1, 16651.9)
ΔH_H	Change in Enthalpy at High Temperatures	$N(67500, 5)$	67500.0 (67499.2, 67500.9)
ΔH_L	Change in Enthalpy at Low Temperatures	$N(-72500, 5)$	-72500.0 (-72500.8, -72499.1)
ϕ	Developtment rate at T_{opt}	$Uniform(0, 2)$	1.341 (0.781, 1.949)
R	Boltzman Gas Constant $\frac{cal}{Kmol}$	Fixed	1.987

Table B.7: The table shows the Ikemoto thermal performance curve and all its parameters, the priors given to them, and the posterior median and highest posterior density intervals. All normal distributions are given as $N(\mu, \tau)$, where $\tau = \frac{1}{\sigma^2}$.
